# The Medicine Habit

**Published:** 1897-08

**Authors:** 


### The Medicine Habit.

One of the most pernicious practices prevailing in this country, and, to a large extent, in all civilized countries, is the habit of medicine-taking. Many people are addicted to the habit of swallowing a drug of some sort for the relief of every physical discomfort which they may happen to experience, without any attempt to remove the cause of the disorder by correcting their faulty habits of life.

The man who does not sleep well at night, instead of finding the cause of his sleeplessness in an indigestible six-o'clock dinner and neglect to take the proper exercise out-of-doors, or some other violations of Nature's laws, swallows some sleep-inducing drug, as bromide, phenacetin, antikamnia, chloral, opium, or, perhaps, a toddy as a "night-cap," until he soon finds that he cannot sleep at all without some hypnotic. Likewise, the man who finds his stomach disordered and his digestion disturbed, instead of seeking to find the cause for the deranged function in the violation of the laws of pietetics—over-eating, too rapid eating, unsuitable combinations of foodstuff, too frequent meals, insufficient exercise, badly cooked and too highly seasoned foods, and similar causes—flies for relief to the drug-store, and doses himself with pepsin and other artificial digestive agents, until after a time his poor stomach becomes, to use the phrase of an eminent European physician, completely "pauperized."

The old system of treating sick men and women by the employment of drugs exclusively was well characterized by the late Dr. Jacob Bigelow, of Boston, as "artificial." It is very gratifying to note that this artificial and irrational method is rapidly giving place to more physiological and rational methods, which seek to effect the cure of the sick by removal of causes, rather than by the antidoting of effects.—*Modern Medicine.*

